# Tellurium

**Published:** 1887-02

**Authors:** George H. Clark

**Affiliations:** Philadelphia


### TELLURIUM.

DR. GEORGE H. CLARK, PHILADELPHIA.

The most marked peculiarities of Tellurium are the otorrhœa, there being an acrid, watery discharge smelling like fish-brine; the condition of the crystalline lens, there being a deposit of a chalky-looking white mass on its anterior surface and pterygium.

The skin symptoms are also prominent, there being ringworms over the whole body, more distinct on the lower limbs.

The other remedies having pterygium are: Calc. carb., Chimaphila, and Zinc.

Allied to it in skin symptoms are: Dulc., Hell., Nat. carb., Phos., Sep.

It is the only remedy in the materia medica having a discharge from the ear smelling like fish-brine. Other remedies having most markedly a discharge from the ear are: Aeth., Alum., Amm. c., Amm. m., Anac., Arund., Asaf., Aurum, Bar. c., Bell., Borax, Bov., Bry., Calc., Calc. phos., Calend., Carbo an., Carbo veg., Caust., Cham., Cist., Colch., Con., Cop., Elap., Ery. a., Graph., Hep., Jug., Kali bich., Kali carb., Kino, Kreos., Lach., Lycop., Meny., Meph., Merc. sol., Merc. viv., Nat. mur., Nit. ac., Petrol., Phos., Psor., Puls., Rhus, Selen., Sep., Sil., Spig., Sulph., Tarent., Tell., Tep., Zinc.

*Ear, discharge from:*
— — — *Acrid:* Tel.
— — — *bloody:* Bar. c., Bell., Bry., Calc., Caust., Con., Elaps., *Graph.*, Lycop., Merc., Mosch., Nit. ac., Petrol., Puls., Rhus, Sep., Sil., Sulph.
— — — *brown:* Anac., Carbo veg., Tarent.
— — — *clear:* Bry.
— — — *excoriating:* Calc. ph.
— — — *fish-pickle*, smelling like: TELL.
— — — *offensive:* Aur., Bov., Carbo veg., Caust., Cist., Ery. a., Hep., Kali bich., Meph., Merc., Merc. cor., Thuja, Zinc.
— — — *purulent:* All. cep., Alum., Amm. c., Amm. m., Arn., *Asaf.*, Aur., Bell., *Borax*, *Bov.*, *Calc.*, Carbo an., Carbo veg., Caust., Cham., Cist., *Con.*, Cop., Gels., Graph., *Hep.*, Kali bich., Kali carb., *Kino*, Lach., Lycop., *Merc.*, Nat. mur., Nit. ac., Petrol., Psor., *Puls.*, Rhus, Sacch., Sep., Sil., Sulph., Zinc.
— — — *discharge from:*
— — — *pus*, smelling like: Bry.
— — — *serous:* Elap, Tarent.
— — — *thick:* Ery. a., Tarent.
— — — *thin:* Cham., Merc., Sep., Sil.
— — — *matter:* *Puls.*, *Merc. sol.*
— — — — *bloody:* Caust., MERC. SOL.
— — — — *brownish:* Anac.
— — — — *offensive:* MERC. SOL.
— — — — *sensation* of: Sil.
— — — — *yellow:* Merc. sol.
— — — *moisture:* Merc. sol., Spig.
— — — — *flesh-colored, offensive:* *Carbo veg.*, Kali carb., Zinc.
— — — — *thick:* *Carbo veg.*
— — — — *yellowish:* Phos.
— — — *mucus:* Bon., Tarent.
— — — *pus:* Acon. l., Aeth., Alum., Arund., Bell., Borax, Calc., Hep., Kali carb., Sacch., Tep., Zinc.
— — — — *bloody:* Ery. a.
— — — — *fetid:* Cist., Meph., Psor., Hep.
— — — — *white:* Ery. a.
— — — — *yellowish green:* Elaps.
— — — — *watery:* Cist., TELL.
— — — — *sensation* of: Acon. Calc., Chr. ac., Cinnab., Der., Graph., Merc. sol. TEL.

